# Effects of proximal fibular osteotomy on stress changes in mild knee osteoarthritis with varus deformity: a finite element analysis

**DOI:** 10.1186/s13018-020-01894-1

**Published:** 2020-09-03

**Authors:** Deng Pan, Lin TianYe, Yang Peng, Xu JingLi, Li HongZhu, Zhao HeRan, Zhang QingWen, Chen LeiLei, Chen ZhenQiu, Wei QiuShi, He Wei

**Affiliations:** 1 The First Clinical Medical College, Guangzhou University of Chinese Medicine, Guangzhou, 510405 Guangdong China; 2 The Lab of Orthopaedics of Chinese Medicine of Lingnan Medical Research Center, Guangzhou University of Chinese Medicine, Guangzhou, 510405 Guangdong China; 3 Department of Joint Orthopaedic, the Third Affiliated Hospital, Guangzhou University of Chinese Medicine, Guangzhou, 510405 Guangdong China; 4 Department of Joint Orthopaedic, the First Affiliated Hospital, Guangzhou University of Chinese Medicine, Guangzhou, 510405 Guangdong China; 5 Department of Joint Orthopaedic, Baoji Hospital of Traditional Chinese Medicine, Baoji, 721000 Shaanxi China

**Keywords:** Proximal fibular osteotomy, Knee osteoarthritis, Varus deformity, Stress, Finite element analysis

## Abstract

**Background:**

Many previous studies lack sufficient quantitative evidences about changes in biomechanical properties of the knee in response to proximal fibular osteotomy (PFO). Therefore, the aim of this study was to compare the preoperative and postoperative effects of PFO on mechanical stresses in the knee joint and provide with a biomechanical basis for PFO in the treatment of mild knee osteoarthritis (KOA) with varus deformity.

**Methods:**

A total of 10 patients suffering mild KOA with varus deformity were enrolled in this study. Their image data from computerized tomography (CT) and magnetic resonance imaging (MRI) were used for finite element models, and PFO models were established. Static structural analysis was carried out using ABAQUS to compare the von Mises stress distribution and values of the maximal von Mises stress of femoral cartilage, meniscuses, tibial cartilages, and tibial plateau before and after surgery.

**Results:**

The stress distribution in the cortical bone of the tibial plateau showed that stresses were transferred from the anterior medial area to the posterior medial area after PFO. Values of the maximal von Mises stress in femoral cartilage, medial meniscus, medial tibial cartilage, and tibial plateau after surgery were significantly lower than the preoperative values, with statistically significant differences (*P* < 0.05). Postoperative values of the maximal von Mises stress of lateral meniscus and lateral tibial cartilage were significantly higher than the preoperative ones, with statistically significant differences (*P* < 0.05).

**Conclusion:**

PFO could reduce the stresses in the medial compartment of the knee joint with stress pathways transferring from the anterior medial area to the posterior medial area of the tibial plateau. Therefore, PFO is recommended for the treatment of mild KOA with varus deformity featuring favorably pain-relieving effects.

**Graphical abstract:**

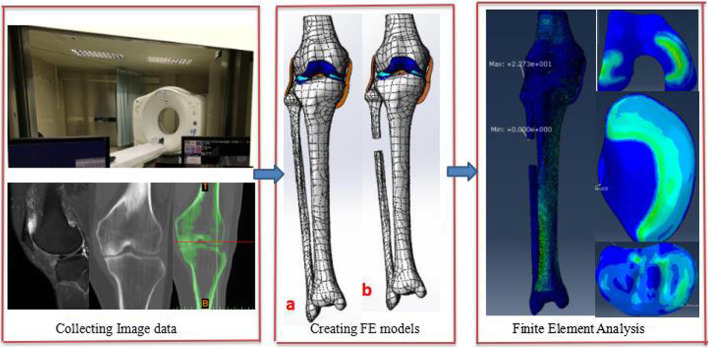

## Introduction

Knee osteoarthritis (KOA) is one of the most common chronic degenerative joint disease in the elderly and one of the leading causes of the loss of ability to walk in the middle-aged and the elderly. Total knee arthroplasty (TKA) is currently recognized as an effective treatment for end-stage KOA [1]. However, after more than 40 years development, a study [2] found that about one third of TKA surgery were currently inappropriate, and it was an “over-medical” problem. Therefore, conservative treatments, such as non-drug therapy, drug therapy, arthroscopic surgery [3], high tibial osteotomy [4], joint traction [5], and platelet-rich plasma (PRP) therapy [6], are still the first choice for early KOA. However, there is still a controversy about conservative treatments because of their various efficacy. Osteotomy is often chosen to treat KOA. High tibial osteotomy (HTO) is a commonly accepted treatment of knee varus deformity resulting from medial femorotibial osteoarthritis (OA) for young and active patients [7]. Faschingbauer et al. [8] have shown that many patients can return to work at the same intensity, and most resume their sporting activities after HTO. However, other researchers have demonstrated that many complications, including nonunion, neurovascular injury, implant irritation, and infection, usually occur after the operation [9].

Recently, PFO has been found to relieve pain and improve knee function of patients with medial compartment KOA. Yang et al. [10] have performed PFO in 156 patients with medial compartment KOA and have found that PFO could improve both the radiographic appearance and the function of varus knee with osteoarthritis and relief long-term pain based on a more than 2-year follow-up. However, there are still widespread controversies about PFO. One main reason is that many previous studies lack sufficient quantitative evidences about changes in biomechanical properties of the knee in response to PFO. Nevertheless, it is difficult to explore the internal stress of bones, because no sensor is sufficiently small to insert into the body without disturbing the responses. By contrast, the finite element (FE) method, a numerical simulation based on the loading condition and measured material properties of the bone, helps to calculate the internal stresses and has been used in many biomechanical studies [11, 12]. Therefore, the aim of this finite element study was to investigate changes in the joint biomechanics in the knee joint after PFO.

## Materials and methods

### General information

A total of 10 patients (consisting of 5 males and 5 females, or 5 right knees and 5 left knees, with the mean age of 53.5 ± 4.04 years) mainly with medial compartment mild KOA complicated with knee varus deformity were randomly selected from our hospital database from February 2017 to June 2017. Patients have to obtain informed consent and sign on informed consent documents before enrolling participants in this study. The mean body mass index (BMI) was 25.38 ± 2.24 kg/m^2^. The main clinical symptoms were medial knee joint pain. Imaging data revealed that the medial compartment of their knee joints was stenotic with mild damage in their cartilages. The X-ray revealed knee varus deformity with the mean varus angle of 7.17 ± 1.32° (Table 1). The patients had no pain relief from non-surgical treatments.

**Table 1:** Clinical characteristics of patients with medial compartment mild KOA and knee varus deformity

Parameter	Values
Age	53.5 ± 4.04 years
Sex	
Female	5
Male	5
BMI	25.38 ± 2.24 kg/m^2^
Mean varus angle	7.17 ± 1.32°
Side	
Left	5
Right	5

Data were presented as mean ± standard deviation or numbers

### Establishment of the knee joint model

**Fig. 1:**
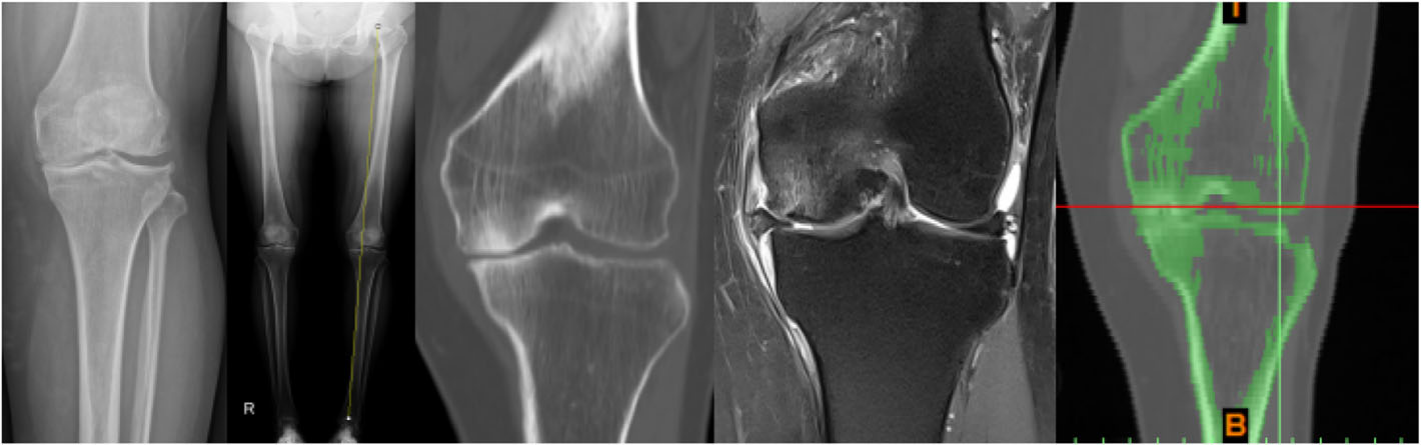
Image data were collected to create a three-dimensional finite element model of the knee joint

**Fig. 2:**
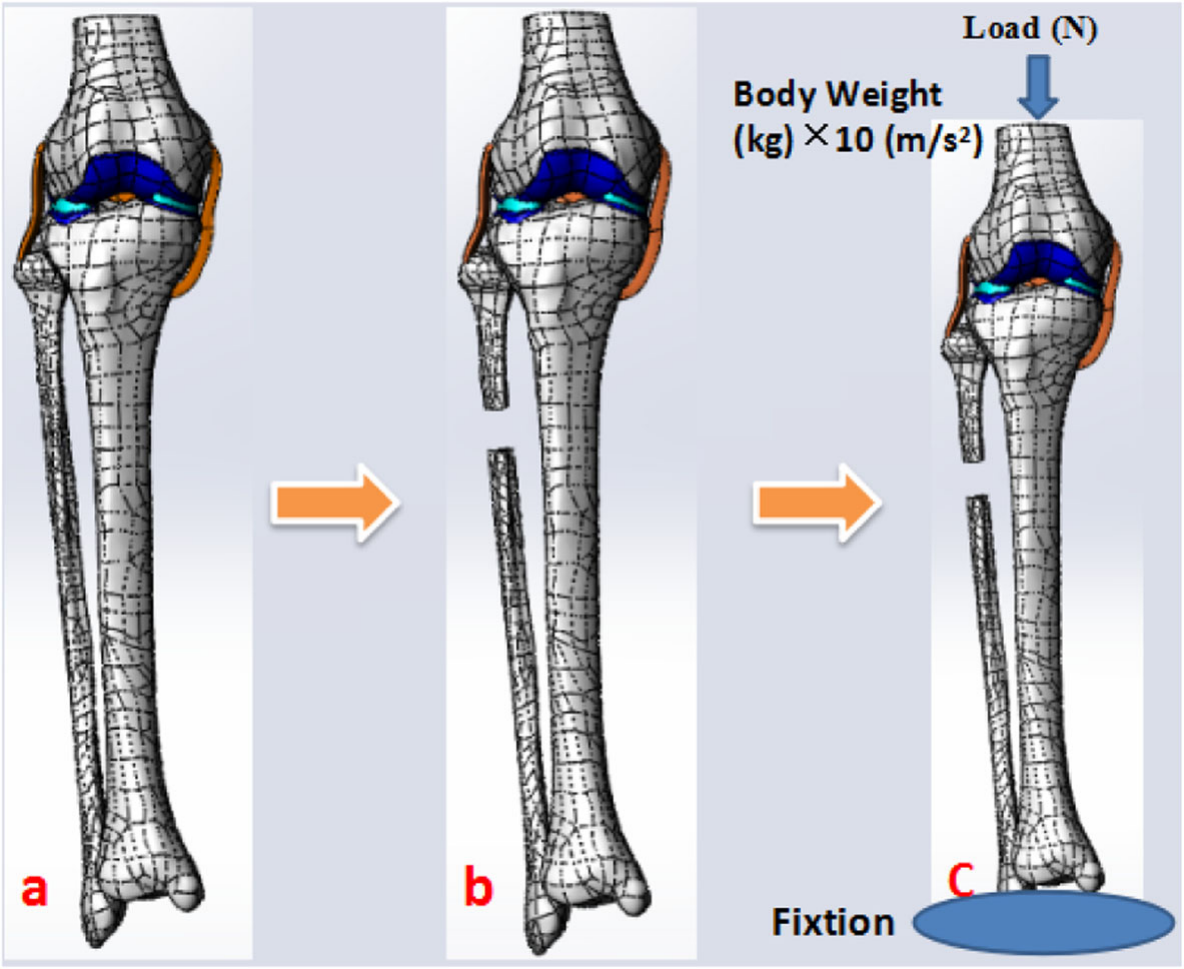
**a** Ten knee models were established. **b** PFO models were established. **c** Loading and boundary condition: a vertical load (*N*), equal to the body weight (kg) × 10 (m/s^2^), was applied to the proximal femur, resulting in a fully constrained condition in the knee

Knee joint CT image data in DICOM format were imported into Mimics software (version 16.0, Materialise NV, Leuven, Belgium). A three-dimensional model of femur, tibia, and fibula was built based on the gray value of the tissue and segmentation of the region. Subsequently, knee joint MRI image data were used to construct the ligament-cartilage model, and the MRI axial T2WI (T2-weighted image) data to construct the anterior-posterior cruciate ligament model and the medial-lateral collateral ligament model. The MRI sagittal T1WI (T1-weighted image) data were adopted to construct the medial-lateral meniscus model, and MRI sagittal T2WI image data the femoral-tibia cartilage model. And then these models were exported as a stl format file (Fig. 1) and were incorporated into Geomagic Studio software (version 11.0, Geomagic Corporation, USA) for smoothing, meshing, and fitting surface processing and also incorporated into the Solidworks software (version 2014, Dassault Systemes, S.A., USA). The ligaments, cartilage, meniscus, tibia, and tibia models were assembled as a knee joint model in the Solidworks software (Fig. 2a). The ligaments were solid element and possessed compression and extension functions.

### Establishment of PFO models

Ten knee joint models were imported into the Solidworks software. About 2–3 cm tibial segments were cut at 6–10 cm below the head of the fibular to generate PFO models (Fig. 2b) [13].

### Material parameter settings

The elastic modulus of cortical bone was set to 16,800 MPa, and Poisson’s ratios to 0.3 [14]. The elastic modulus and Poisson’s ratio of the trabecular bone were set to 840 MPa and 0.2 [15]. The menisci and cartilage were modeled as linear elastic materials due to the incompressible nature of the cartilage tissue under short loading times as A.R. Hopkins study [16]. The elastic modulus and Poisson’s ratio of the menisci were 80 MPa and 0.3, and those of the cartilage were 12 MPa and 0.45, respectively. The elastic modulus and Poisson’s ratio of the ligament were 390 MPa and 0.4 [17]. The two indices of various structural materials were shown in Table 2.

**Table 2:** Material properties of various components in the model

Items	Elastic modulus (MPa)	Poisson’s ratio
Cortical bone	16,800	0.3
Cancellous bone	840	0.2
Cartilage	12	0.45
Menisci	80	0.3
Ligament	390	0.4

### Boundary conditions and loading force

During simulation, the distal end of the tibia and fibula were completely fixed, while the femur was completely unconstrained except for the flexion angle, which was fixed in full extension [18]. The femur cartilage and the femoral cortical bone were placed in a binding relationship. Below the meniscus and the lower end of the tibia were also placed in a binding relationship. The ligament and the bone were placed in a binding relationship. The upper part of the meniscus and femur cartilage were set in a frictional relationship, and the coefficient of friction was 0.2 [14]. A vertical load (*N*), equal to body weight (kg) × 10 (m/s^2^), was applied to the proximal femur of each patient by Abaqus 6.10.1 (Simulia, Rhode Island, USA) (Fig. 2c) [18].

### Mesh convergence

Sensitivity analyses on mesh density were performed to verify that the model predictions were not affected by mesh refinement. The element size of knee substructures was varied to yield six different mesh resolutions, by keeping the very refined mesh as the reference for comparison (Table 3). The peak joint contact pressure predicted by cases A–E were compared with those predicted by the reference case, and the cases within 5% of the reference case were considered as accurate. Case C was found to be optimal, as it requires less computing power while maintaining prediction accuracy of 96% with respect to the reference case model. The predictions by cases D and E were very inaccurate (> 5%).

**Table 3:** Sensitivity analyses on mesh density for different knee substructures

Case(s)	Element size (ES) and number of elements (NOE)								% change in peak cartilage contact pressure
	Femoral cartilage		Tibial cartilage		Medial meniscus		Lateral meniscus		
	ES (mm)	NOE	ES (mm)	NOE	ES (mm)	NOE	ES (mm)	NOE	
Reference	0.75	35040	0.5	40315	0.5	8725	0.5	12540	–
Case A	0.75	35040	0.5	40315	1	1375	1	1728	1.96
Case B	0.75	35040	1	6092	1	1375	1	1728	2.87
Case C	1	13780	1	6092	1	1375	1	1728	3.98
Case D	1.5	3170	1.5	1260	1.5	435	1.5	495	9.46
Case E	2	1835	2	837	2	228	2	258	14.36

### Evaluation criteria

Firstly, the von Mises stress distribution of femoral cartilage, meniscuses, tibial cartilages, and tibial plateau was observed before and after PFO. Secondly, preoperative and postoperative values of the maximal von Mises stress of femoral cartilage, meniscuses, tibial cartilages, and tibial plateau were compared.

### Statistical analyses

Each statistical analysis was performed using IBM SPSS Statistics for Windows, Version 21.0 (IBM Corp, Armonk, NY, USA). Measurement data were expressed as mean ± standard deviation, and paired *t* test was used for comparisons. A *P* value of < 0.05 was considered as statistically significant.

## Results

### The von Mises stress distribution in different parts of the knee joint before and after PFO

**Fig. 3:**
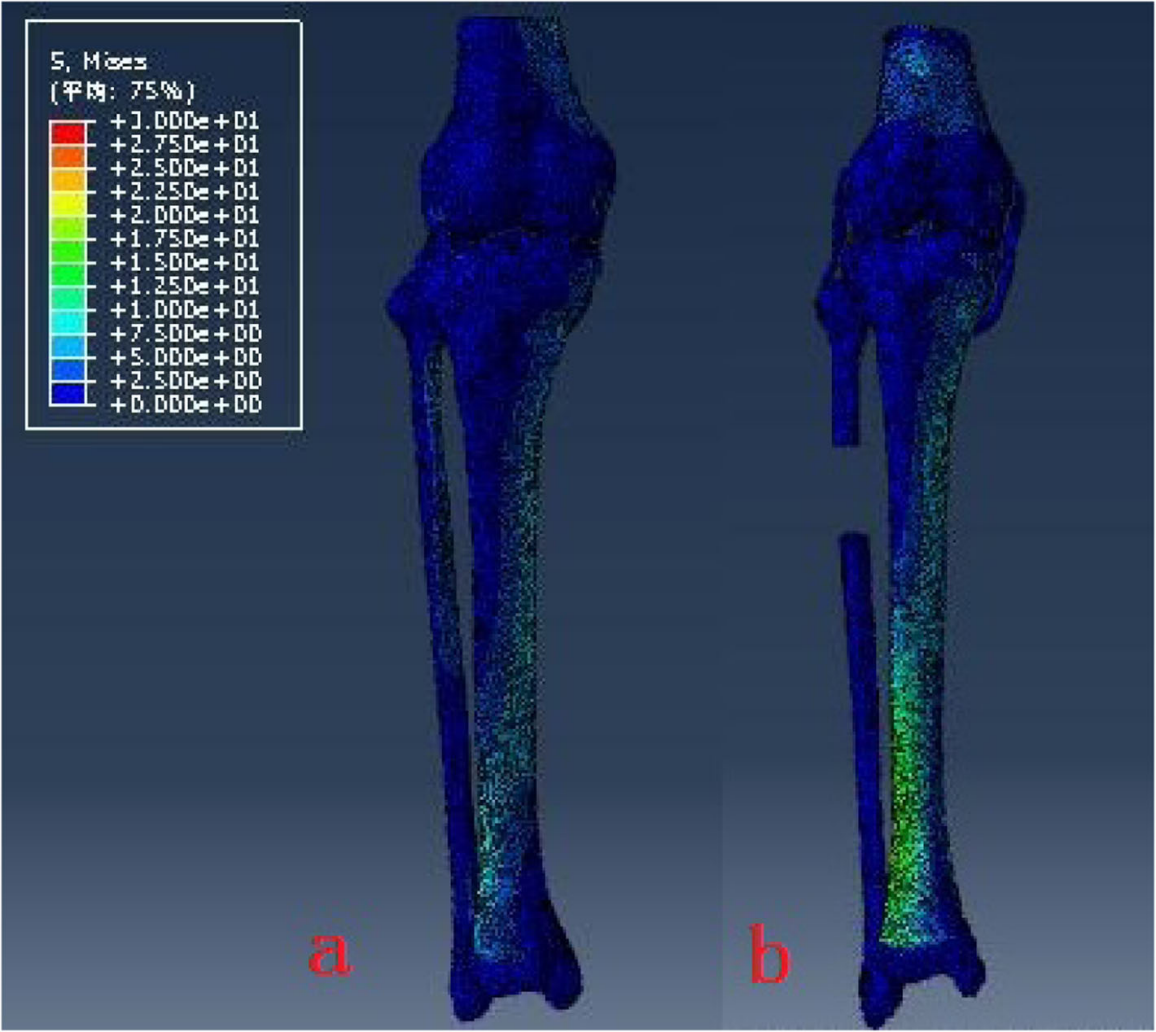
The stress pathways in different knee-joint models before and after PFO

**Fig. 4:**
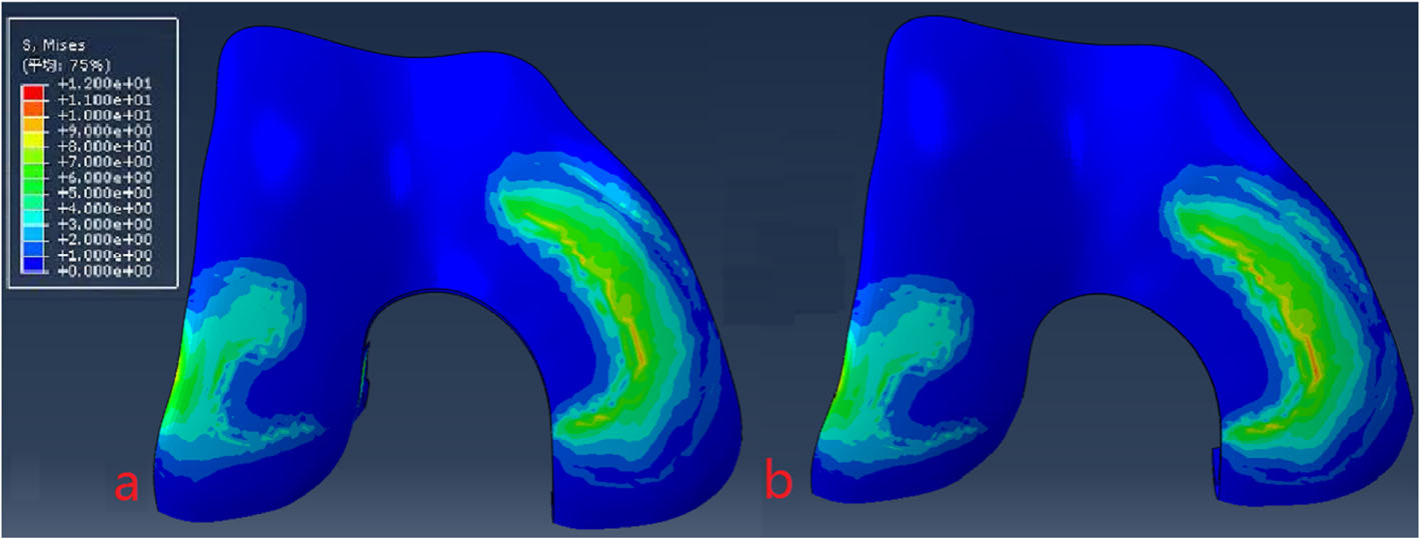
The von Mises stress distribution the femoral cartilage before (**a**) and after (**b**) PFO

**Fig. 5:**
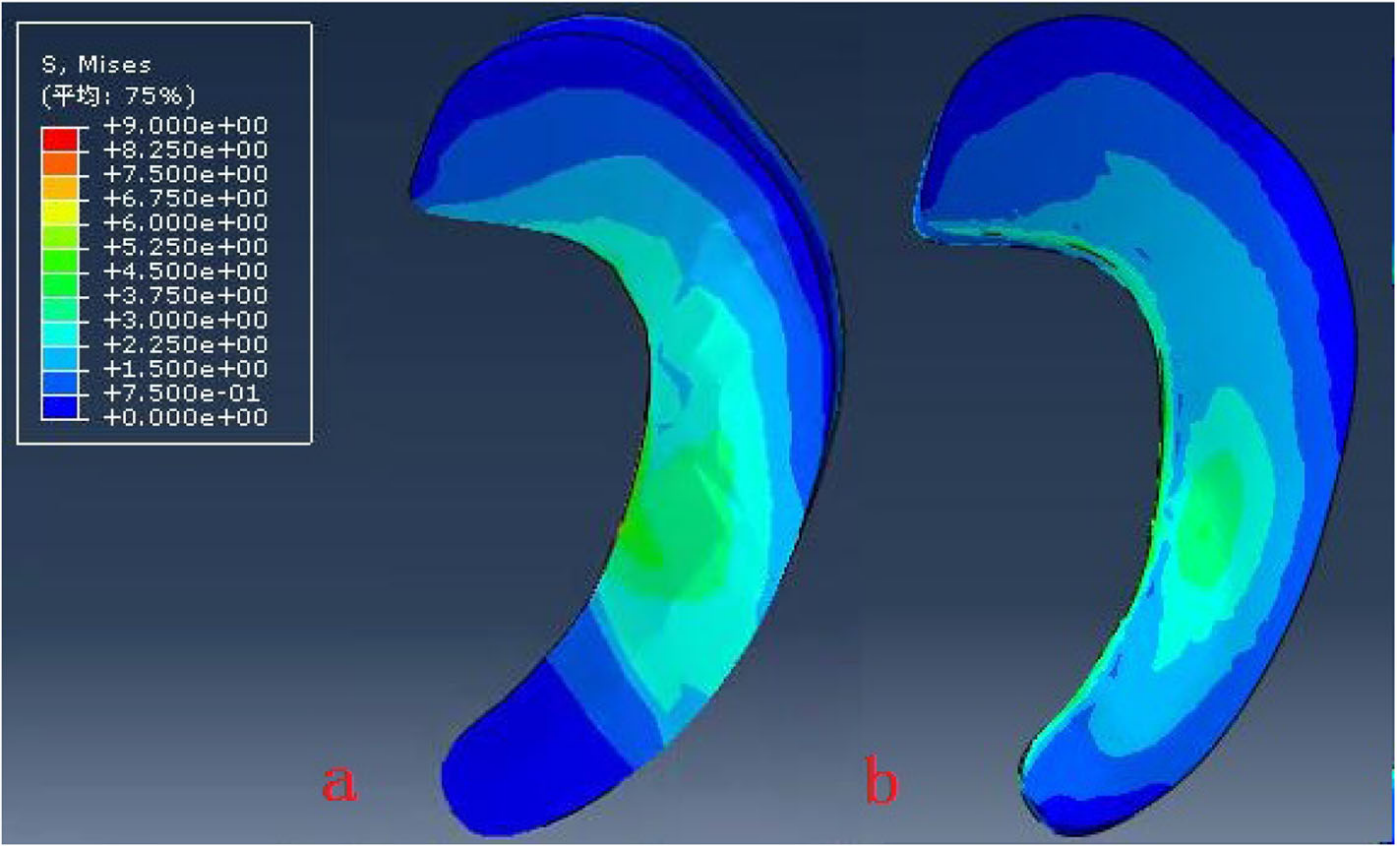
The von Mises stress distribution the medial meniscus before (**a**) and after (**b**) PFO

**Fig. 6:**
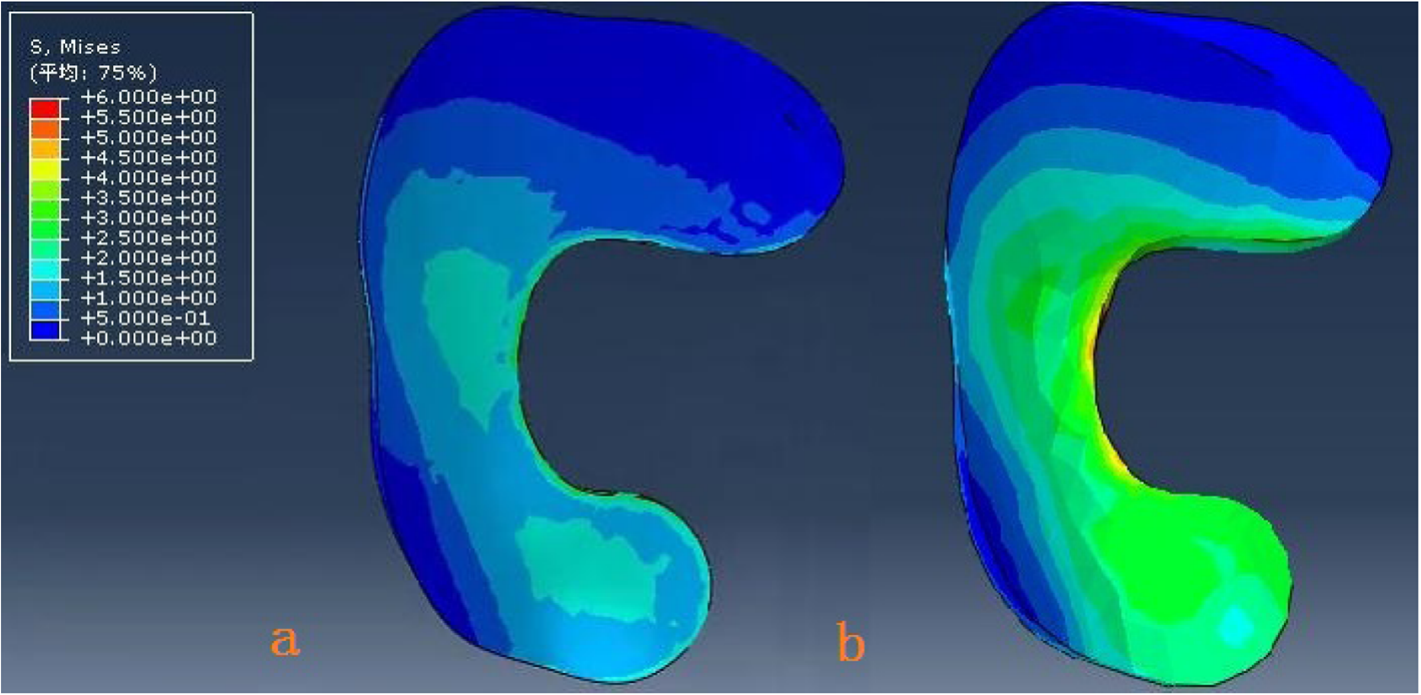
The von Mises stress distribution the lateral meniscus before (**a**) and after (**b**) PFO

**Fig. 7:**
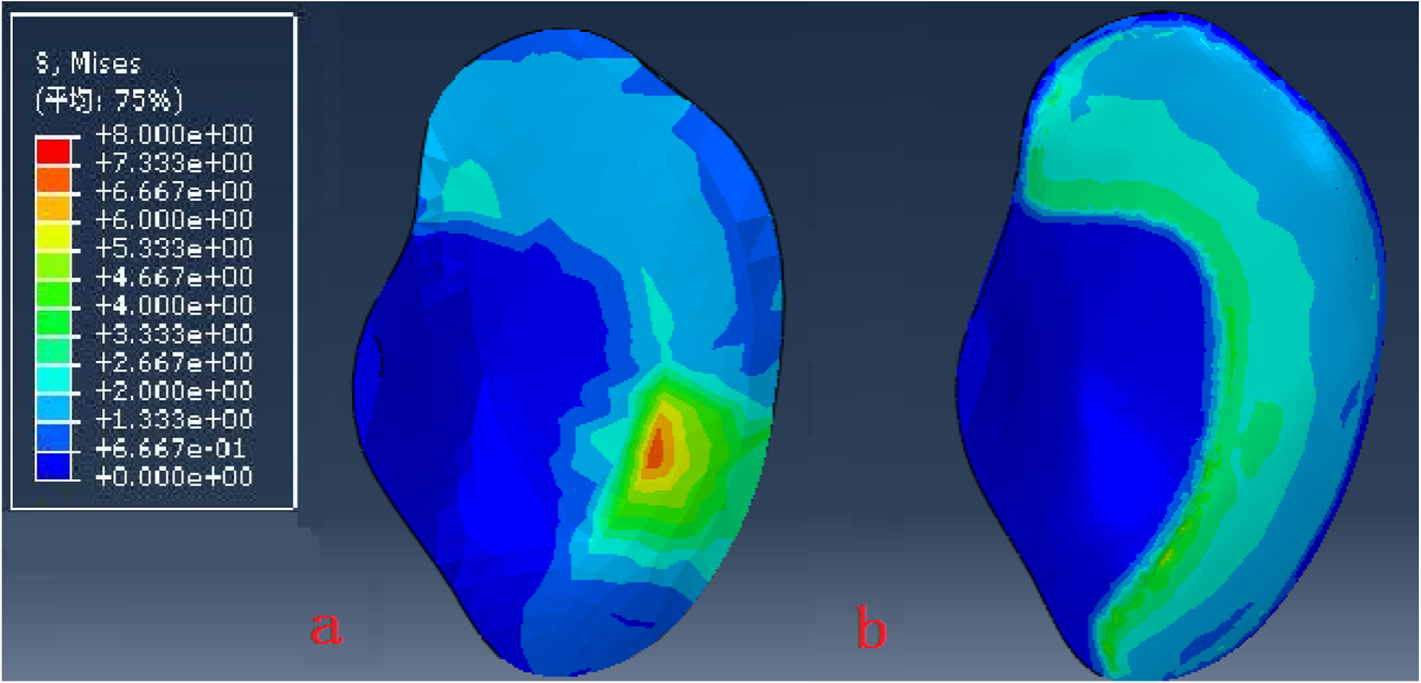
The von Mises stress distribution the medial tibial cartilage maximum before (**a**) and after (**b**) PFO

**Fig. 8:**
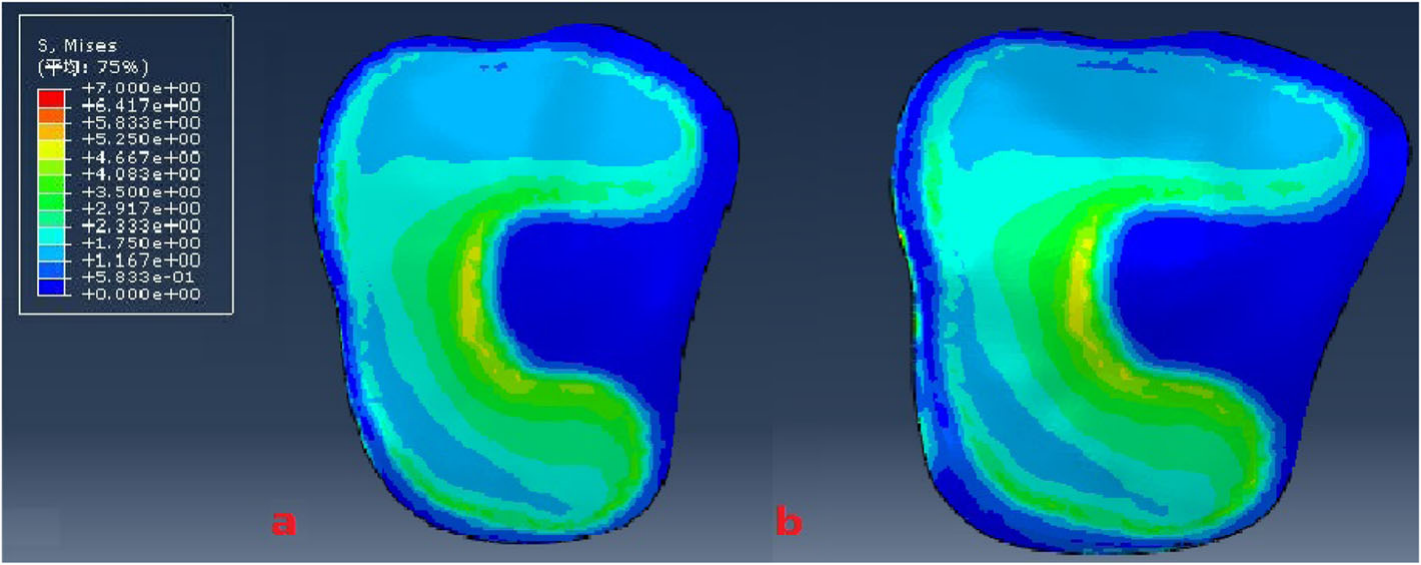
The von Mises stress distribution the lateral tibial cartilage before (**a**) and after (**b**) PFO

**Fig. 9:**
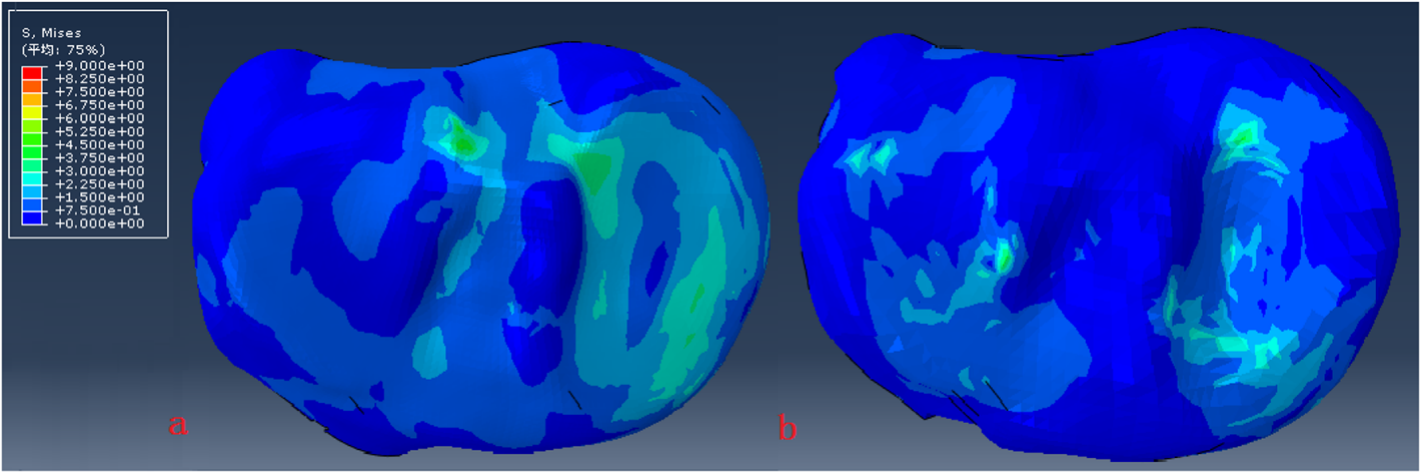
The von Mises stress distribution and the cortical bone of tibial plateau before (**a**) and after (**b**) PFO

The model of the before PFO contains 53,256 nodes and 1,73,214 elements, and the model of after PFO has 49,359 nodes and 1,12,258 elements. The von Mises stress distribution in the knee reflected changes in stress pathways before and after PFO. Prior to PFO, both the tibia and fibula were loaded while the tibia was loaded much more than the fibula. Before the PFO, both the tibia and fibula were loaded while the tibia was loaded much more than the fibula. However, only the tibia was loaded after the surgery (Fig. 3). Stress distribution patterns of femoral cartilage, meniscuses, tibial cartilages, and tibial plateau before and after PFO were illustrated in finite element stress nephogram. It was found that the area of stress distribution in the medial femoral cartilage was larger than that in the lateral femoral cartilage. Stress in the femoral cartilage was reduced after PFO (Fig. 4). The area of stress distribution in the medial meniscus was larger than that in the lateral side preoperatively. After PFO, stress in the medial meniscus decreased (Fig. 5) but that in the lateral side increased (Fig. 6). Furthermore, stress in the medial tibial cartilage was reduced (Fig. 7) and that in the lateral tibial cartilage increased after the surgery (Fig. 8). The maximum stress loaded on the medial side of the cortical bone after PFO was lower than that before the surgery. Before the PFO, the stress on the medial side of the tibial plateau was greater. However, the medial stress on the tibial plateau decreased and the lateral stress increased after the PFO. The stress distribution in the cortical bone of the tibial plateau showed that stress was transferred from the medial area to the lateral area after the surgery (Fig. 9).

### Quantitated maximum von Mises stress of different knee joint structures before and after PFO

Values of the maximum von Mises stress of 10 PFO models were compared preoperatively and postoperatively. It was found that the postoperative values of the maximum stress of femoral cartilage, medial meniscus, medial tibial cartilage, and tibial plateau were significantly lower than those before PFO with statistically significant differences (*P* < 0.05). The postoperative values of the maximum stress of lateral meniscus and lateral tibial cartilage were significantly higher than those before PFO with statistically significant differences (*P* < 0.05) (Table 4).

**Table 4:** Values of the maximum von Mises stress of different knee joint structures before and after PFO

Parts	Pre (*n* = 10)	Post (*n* = 10)	*P* value
Femoral cartilage (MPa)	11.87 (0.13)	11.49 (0.14)	< 0.001
Medial meniscus (MPa)	9.21 (1.36)	7.90 (1.03)	0.003
Lateral meniscus (MPa)	4.51 (0.55)	5.63 (0.51)	< 0.001
Medial tibial cartilage (MPa)	7.73 (0.63)	6.90 (0.27)	0.001
Lateral tibial cartilage (MPa)	5.88 (0.19)	6.72 (0.14)	< 0.001
Tibial plateau (MPa)	9.03 (1.34)	7.94 (0.99)	0.002

*Pre*, values of the maximum von Mises stress of different knee-joint parts before PFO. *Post*, values of the maximum von Mises stress of different knee-joint parts after PFO. The corresponding values were expressed as means (standard deviations) in the table above

## Discussion

PFO is considered as a new treatment of KOA with varus deformity, featuring simple operation, low cost, and quick recovery with good short-term efficacy compared with other procedures. It is easy to promote in primary hospitals. Currently, however, this surgical method has become a moot point because of insufficient biomechanical evidences to support its precise efficacy [19]. In this study, a three-dimensional finite element analysis has been performed to establish an individualized three-dimensional finite element model of KOA with knee varus deformity based on CT and X-ray imaging data of the patients. After we collected and analyzed the pre- and post-operative biomechanical data of the knee joint in patients with KOA and varus deformity, it was revealed that the stress concentration area shifted to the lateral compartment after PFO, so stress in the medial compartment of the knee joint was reduced.

KOA with varus deformity is a common clinical disease with the main manifestations of pain in the medial compartment and knee joint function limitation. It brings about serious impacts on the daily life of elderly patients, and even can cripple some with severe KOA. Despite the favorable efficacy of TKA for the treatment of KOA with varus deformity [20], people have encountered problems, such as loose knee prostheses, prosthetic infections, dislocations, and bone resorption [21], in its clinical practices. So, researchers have refocused on safe and effective conservative treatments of knee arthritis and difficulties in their applications to avert these issues. A clinical study [22] has showed that PFO can improve both the radiographic appearance and knee function of patients with KOA. However, the mechanical evidences behind its efficacy remains insufficient. Some cadaveric experiments have uncovered that PFO can reduce stresses in the medial compartment of the knee. Yazdi et al. [23] have cut off the 20-mm long proximal fibulae in 6 cases and have found that the overall knee joint stress has been reduced, revealing a more significant decrease in the medial compartment stress with the maximum decrease of 21.6% and an increase in the lateral compartment stress with the maximum increase of 12.9%. In our finite element experiment, PFO changed the stress distribution in the knee joint and reduced the medial compartment stress especially in KOA patients. A clinical study concludes that the fibula supports one sixth of the body weight [13]. Therefore, PFO mainly helps rebalance or redistribute the load on the lateral and medial tibia plateau after surgery. In our finite element experiment, von Mises stress distribution in the knee before and after PFO illustrated variations of stress pathways in the knee. In particular, stress in the tibia significantly changed after the surgery. Another possible mechanism is the nonuniform settlement proposed by Yang et al. [10]. As people get older, they believe that the bone density of the knee tibial plateau reduces over time. For adapting to these changes, there may be no strong bone structural to support the medial side of the tibial plateau, but sufficient fibular support on the lateral side. Therefore, the rate of settlement inside and outside the tibial plateau becomes inconsistent. More medial-side settlements in the tibial plateau can result in knee varus deformity. Zhang et al. [13] have reported that the femoral condyle can medially shift during walking and sports with the presence of the transverse shear forces due to the tibial plateau slope arising from a nonuniform settlement. The support force on the lateral tibial plateau can be removed after PFO, which will relieve the medial compartment pressure and the resulting knee pain. Moreover, a three-dimensional gait analysis study demonstrated that PFO was beneficial for delaying the progression of knee arthritis [19]. This study provides with a significant quantitative approach for analyzing knee adduction moment in patients with KOA who present a 10–20% reduction in the overall peak knee adduction moment after PFO. In addition, a study of bone mechanics has reported that medial forces are reduced by 24%/30% on average during walking with the knee brace with mild (4°/8°) valgus adjustment [24]. Therefore, we speculate that the soft tissue of the knee joint can be rebalanced after PFO. Specifically, as the fibula tightly connects with the tibia prior to PFO, the lateral soft-tissue tension remains low. Once PFO has been performed, the head of the fibula will have greater freedom. After PFO, the soft tissue of the knee joint will form a new balance. The lateral ligaments and muscles of the knee joint will tighten while the medial side will relax. The lateral soft tissue of the knee will pull up the femoral condyle, reducing the tension of the medial tibial plateau. However, PFO also presents some limitations in correcting the alignment of the lower limb. A clinical study has reported that PFO is merely suitable for patients with mild varus deformity of the knee (≤ 5°) and has recommended applications of restorable spacers and four-point support plates to correcting alignment of lower limb in terms of severe varus deformity (≥ 5°) [25]. In our study, the average knee varus angle in KOA patients was 7.17 ± 1.32°, and post-PFO values of the maximal von Mises stress of femoral cartilage, medial meniscus, medial tibial cartilage, and tibial plateau in all patients were significantly lower than those before the surgery.

The precise location of the osteotomy is critical for performing PFO. The integrity of the fibula, particularly the 6-cm level above the distal fibula, is essential for maintaining the stability of ankle joint [26]. Besides, injury to the peroneal nerve and its branches is the most common complication of PFO because it is anatomically close to the fibula [27]. The superficial peroneal nerve bypasses the lateral border of the fibula, and about one third of the deep peroneal nerve is located at the leading edge of the fibula [28]. Hence, it has been concluded that a position about 6–10 cm from the tip of the fibula is the best site for fibula osteotomy [9]. In our study, a 2- to 3-cm tibial segment was incised at the 6- to 10-cm level below the head of the fibular during PFO, and there was no abnormal stress in ankle joints. HTO is an effective treatment for mild KOA with varus deformity, however, followed by a series of complications encompassing mechanical failure, delayed union, deep vein thrombosis, etc. [29]. Similar to HTO, TKA also can bring about complications incorporating wound infection, peripheral nerve injury, osteolysis, periprosthetic fracture, etc. [30]. Therefore, the advantages of PFO are evident, featuring straightforward, affordable, and quicker recovery with less operative trauma and fewer complications than the aforesaid treatments.

The main innovation of this study is constructing a finite element model of the knee joint based on data fusion in multimodal CT and MRI imaging. The validity of the models has been verified by mechanical analysis. The results of compressive stress of the knee under static stance simulation were compared to previous study. Nie et al. [19] research showed that the mean quantitative stress value of the anterior and posterior tibial plateau was 10 MPa. The compressive stress (9.03 MPa) on the tibial plateau of our intact knee model was slightly lower than the previous studies. However, the loads of studies were not the same and, as a result, the compressive stress remained different. Besides, the differences were not significant and were in the same order of magnitude. Therefore, we thought the results of stress from our finite element simulation is in agreement with the previous study. This study provides with the biomechanical mechanism of PFO in treating patients with mild KOA using finite element stress analysis. As the present study is based on the finite element analysis on simulated reconstruction models using CT and MRI image data, limitations of our study were that the material properties of each model were set as a uniform value, instead of the real values. The variation of the material properties between the subjects were not considered. Moreover, we simplified the knee model and PFO details and ignored the impacts from surrounding muscles. The actual surgical procedure might be more complicated than the experimental one. Thus, further studies of PFO require a larger sample of clinical research applications.

## Conclusion

By establishing a finite element model, we have found that PFO can reduce stress in the medial compartment of the knee joint and make stress pathways in the tibial plateau transfer from the anterior medial area to the posterior medial area. That is why PFO usually reduces pain in patients with mild knee arthritis. Therefore, PFO is recommended for treating mild KOA with varus deformity.

## Data Availability

All the data will be available upon motivated request to the corresponding author of the present paper.

## Abbreviations

PFO: Proximal fibular osteotomy
KOA: Knee osteoarthritis
CT: Computed tomography
MR: Magnetic resonance
BMI: Body mass index
DICOM: Digital imaging and communications in medicine
